# High-throughput evaluation of cardiac-specific promoters for adeno-associated virus mediated cardiac gene therapy

**DOI:** 10.1038/s41434-025-00553-6

**Published:** 2025-07-19

**Authors:** Dhanya Ravindran, Renuka Rao, Juan Mundisugih, Tracy Titus, Shinya Tsurusaki, Cindy Y. Kok, Fairooj N. Rashid, Sindhu Igoor, Yasuhito Kotake, Saurabh Kumar, James J. H. Chong, Ian E. Alexander, Leszek Lisowski, Eddy Kizana

**Affiliations:** 1 Centre for Heart Research, The Westmead Institute for Medical Research, The University of Sydney, Westmead, NSW Australia; 2 Faculty of Medicine and Health, The University of Sydney, Sydney, NSW Australia; 3 Department of Cardiology, Westmead Hospital, Westmead, NSW Australia; 4 Translational Vectorology Research Unit, Children’s Medical Research Institute, Westmead, NSW Australia; 5 Gene Therapy Research Unit, Children’s Medical Research Institute and Children’s Hospital at Westmead, The University of Sydney and Sydney Children’s Hospital Network, Westmead, NSW Australia; 6 Discipline of Child and Adolescent Health, Faculty of Medicine and Health, The University of Sydney, Westmead, NSW Australia; 7 Australian Genome Therapeutics Centre, Children’s Medical Research Institute and Sydney Children’s Hospitals Network, Westmead, NSW 2145 Australia; 8 Laboratory of Molecular Oncology and Innovative Therapies, Military Institute of Medicine - National Research Institute, Warsaw, Poland

**Keywords:** Gene delivery, Gene expression

## Abstract

The selection of an appropriate promoter is important to the design and optimisation of adeno-associated viral (AAV) vector-based cardiac gene therapies. The expression cassette design can impact efficacy and safety of the vector. This study is the first to use a novel AAV barcode-seq method for the simultaneous evaluation of a panel of cardiac-specific promoters in a high-throughput manner. Functional analyses of our cardiac promoter kit packaged in three different capsids were performed using neonatal rat ventricular myocytes (NRVM), human iPSC-derived cardiomyocytes (hiPSC-CMs), HuH7 hepatocellular carcinoma cells, as well as mouse, rat, sheep and pig models. The cardiac troponin T (cTnT) promoter showed the most promise overall as a cardiac-specific promoter across all cardiac models tested. The results validate the barcode-seq technique as a powerful and versatile approach that enables high-throughput, quantitative analysis of various expression cassettes in commonly used models of cardiac gene therapy.

## Introduction

Several gene therapy products using recombinant rAAV have advanced to clinical use [1–3]. Similar outcomes have not been achieved for cardiac disease due to the limitations of the gene therapy technology [4, 5]. For effective cardiac transduction, the ability to precisely deliver the genetic payload and achieve robust transgene expression in the heart without triggering unwanted off-target effects are the most critical features when choosing an AAV capsid and designing expression cassettes for clinical applications. While considerable effort has been dedicated to modifying AAV vector capsids to enhance their delivery efficiency and tissue specificity [6–8], less attention has been given to optimising the promoter for controlling gene expression. Ubiquitous promoters are frequently chosen to reach potent expression, with over 50% usage reported in 106 clinical trials [9]. On the contrary, the use of cell- or tissue-specific promoter can regulate precisely localised gene expression to minimise off-target effects and related immune-mediated toxicity.

Several research groups have compared the performance of different AAV vector constructs by testing them individually, typically using fluorescent reporter expression as a measure of transduction efficiency [10–14]. While these approaches can provide reliable results, the significant labour involved limits the number of vectors that can be compared simultaneously, particularly when in vivo animal models are employed. Recent advancements have introduced high-throughput approaches [15–18] based on next-generation sequencing (NGS), coupled with custom bioinformatic analysis pipelines, as alternative methods for detecting vector genomes in competitive transduction assays.

In this study, we examined the power of this barcoded technology to study the performance of six cardiac-specific promoters assembled into a cardiac promoter kit. These kits were packaged in commonly used AAV capsids including AAV2, 6 and 9. They were evaluated on NRVM, hiPSC-CM, and HuH7, as well as in vivo in mouse, rat, sheep and pig models. The results validate the NGS-based testing protocol as a powerful tool for screening and selecting the top cardiac promoter candidates for individual testing in relevant in vitro and in vivo cardiac models.

## Materials and methods

### Animal ethics statement

Animal procedures were conducted in accordance with and approved by the Western Sydney Local Health District Animal Ethics Committee and were performed in accordance with the National Health and Medical Research Council (NHMRC) Code for the care and use of animals for scientific purposes. Protocol approval numbers were as follows: Isolation of neonatal rat ventricular myocytes (NRVMs) (4332.08.20), rAAV transduction of mice, rats (4348.05.21), pig and sheep (5162.09.19).

### Creation of cardiac-specific barcoded rAAV promoter kit

AAV vectors containing ITR2-CMV-βglobin intron-eGFP-N_6_Barcode (BC)-WPRE-ITR2 with 18 unique BCs were created as detailed in Supplementary Methods S1 and Table S1.

### Differentiation of hiPSC cardiomyocytes

SCVI 8 hiPSC line obtained from Prof. Joseph Wu (Stanford Cardiovascular Institute) was used in this study. Cardiomyocyte differentiation was carried out as described [19] in Supplementary Methods S2.

### Isolation of neonatal rat ventricular myocytes (NRVMs)

Primary cultures of NRVMs were prepared by enzymatic digestion of ventricles obtained from neonatal (2–3 day old) Sprague-Dawley (SD) rats, as previously described [20].

### Flow cytometry to determine cardiomyocyte purity

hiPSC-CM (D15 post differentiation) and NRVM (D2 post isolation) were stained with cTnT antibody and analysed by flow cytometry as described in Supplementary Methods S3.

### Transduction of hiPSC-CM

Cells were transduced with the rAAV promoter kit packaged in AAV2 or 6, at MOI 1000, in RPMI 1640 medium (Thermo Fisher Scientific, 21870076) and B27 supplement (Thermo Fisher Scientific, 17504-001). On D5 post transduction, cells were imaged using the Zeiss Axiovert 200 M Live Cell Imaging Microscope (Zeiss, Oberkochen, Germany), then harvested for nucleic acid extraction using the AllPrep DNA/RNA Micro Kit (QIAGEN, Hilden, Germany, no. 80284).

### Transduction of NRVM

Following the switch to maintenance media (M199 + 2% FBS), cells were transduced with the rAAV promoter kit packaged in AAV6 or 9, at an MOI of 1000 on D2. Cells were collected on D5 post transduction, and DNA /RNA was extracted using the AllPrep DNA/RNA Micro Kit.

### Transduction of HuH7 cells

HuH7 (Hepatocellular carcinoma) cells (Cell Bank, Children’s Medical Research Institute, Sydney, Australia) were maintained in 10% FBS containing DMEM (ThermoFisher Scientific, no 11995065). Cells were plated at 2 × 10^5^ cells/well in a 24 well plate. 24 h later cells were transduced with the rAAV promoter kit packaged in AAV2 or 6 at an MOI of 1000. On D5 post transduction, cells were imaged using the Zeiss Axiovert 200 M Live Cell Imaging Microscope, then harvested for nucleic acid extraction using the AllPrep DNA/RNA Micro Kit.

### Recombinant AAV production and transduction to assess performance of individual promoters

Constructs of the overall top 3 cardiac-specific promoters (αMHC, NCX and cTnT) as well as controls CMV and LSP were packaged in AAV6. rAAV production, purification, concentration and titration was carried out as described [20]. Transduction and flow cytometry are described in detail in Supplementary Methods S3, S4 and S5.

### Cardiac transduction of mouse and rat

C57Bl6 mice (6–8-week males) and SD Rats (6–8-week males) were injected with 1×10^11^ vg and 5×10^11^ vg respectively of the rAAV promoter kit packaged in AAV6 and 9, via the tail vein. After 4 weeks, animals were sacrificed. Livers and hearts were collected for DNA/RNA extraction using the AllPrep DNA/RNA Mini Kit (QIAGEN, Hilden, Germany, no. 80204), which were then used for subsequent NGS.

### Cardiac transduction of pig and sheep

Adult male Merino cross sheep (~50 kg) and female Landrace pigs (~20 kg) were injected intramyocardially with the rAAV promoter kit packaged in AAV6 at 5 × 10^11^ vg/animal and AAV9 at 1 × 10^12^ vg/animal following an iodine contrast pretest to confirm the injection site. Viral vectors were delivered into the right ventricle (RV) septum in pigs and apex in sheep, through a steerable NOGA MyoStar injection catheter (Biological Delivery System, Cordis, Johnson & Johnson, USA) under fluoroscopic and intracardiac echocardiography guidance. After 2 weeks, animals were sacrificed. Hearts were collected for DNA/RNA extraction (RNeasy Midi Kit, QIAGEN, Hilden, Germany, no. 75144) which were then used for subsequent NGS.

### Next-generation sequencing analysis

For NGS analysis, DNA/RNA samples from hiPSC-CM, NRVM, HuH7, mice, rats, sheep and pigs were processed and analysed as described [19]. Detailed workflow for analysis using Snakemake to process reads and count barcodes, and Python script to identify barcodes corresponding to AAV variants were as described previously [21]. Next-generation sequencing reads from DNA and RNA populations were normalised to the reads from the pre-transduction mix. Transduction efficiency was determined using Expression Index (EI) which was derived from relative proportions of barcode reads at the level of gene expression (mRNA/cDNA) normalised to cell entry (gDNA).

### Statistical analysis

Continuous variables were presented as means. All statistical analyses and data visualisation were conducted using R Statistical Software version 4.1.2 (R Foundation for Statistical Computing, Vienna, Austria). The Shapiro-Wilk test revealed non-normal distribution of the data, necessitating non-parametric statistical approaches. Consequently, we employed the Kruskal-Wallis test to assess overall differences between groups, followed by Dunn’s post-hoc test with Bonferroni correction for multiple comparisons using the ‘dunn.test’ R package. For all analyses, statistical significance was established at *p* < 0.05 and denoted as *p < 0.05 in results. Visualisation of results was accomplished through boxplots generated using the ‘ggplot2’ package. These boxplots compared promoters both as categorical groups (CMV, cardiac-specific promoters [CSPs], and LSP) and within the cardiac-specific promoter subset. The study’s summary was synthesised using a bubble plot format, with both the underlying datasets and analytical code available in Supplementary Tables S2 and S3, respectively.

## Results

### NCX and cTnT were the top performing cardiac-specific promoters in commonly used cardiomyocyte-enriched cell culture models

**Fig. 1:**
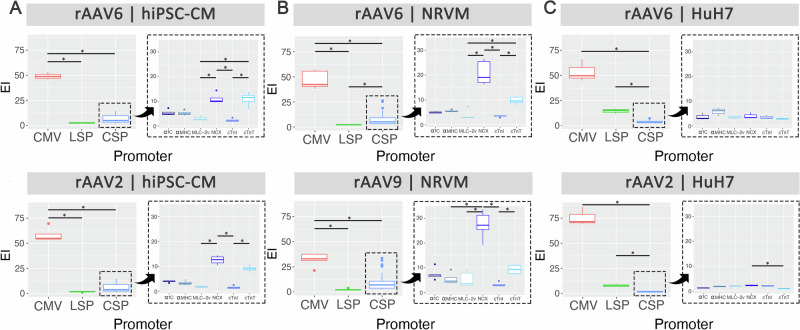
Competitive transduction assay to compare efficiency of barcoded rAAV promoters in vitro. **A** hiPSC-CM, **B** NRVM and **C** HuH7 were competitively transduced with a rAAV promoter kit packaged in AAV2, 6 and 9 at MOI 1000 (*n* = 4 hiPSC-CM, *n* = 5 NRVM, *n* = 3 Huh7). For each capsid and cell type combination, results on the left are displayed as boxplots comparing performance of ubiquitous promoter CMV, cardiac-specific promoters (CSP) and liver-specific promoter (LSP), and on the right as boxplots comparing performance of cardiac-specific promoters only. Statistical analyses were performed using the Kruskal-Wallis test followed by Dunn’s post-hoc test with Bonferroni correction (*p < 0.05).

We compared the performance of cardiac-specific promoters in vitro using the barcode-seq method [15]. These promoters were vectorised and compared with the ubiquitous CMV promoter and a liver-specific promoter (LSP) using a competitive transduction assay. Each vectorised promoter was separately used to package a recombinant AAV-GFP-driving expression cassette identified by a unique barcode, which allowed for the mixing of vectors at an equimolar ratio and used to transduce cells simultaneously. Figure 1 shows the results of hiPSC-CMs, NRVMs and HuH7 transduced with the custom kit packaged in two capsids per cell type. Recombinant AAV2 and AAV6 were used for hiPSC-CM and HuH7, while rAAV6 and rAAV9 were used for NRVM. Transduction efficiency was determined using the EI.

As expected, the ubiquitous promoter CMV performed the best in all in vitro models (Fig. 1A–C, left panels). The grouped data in Fig. 1A and B also show that the cardiac-specific promoters outperformed LSP in the cardiomyocyte-enriched cells irrespective of the species, while LSP performed significantly better than the cardiac-specific promoters in HuH7, a liver-specific cell line (Fig. 1C). Within the cardiac-specific promoters, NCX and cTnT significantly outperformed the rest irrespective of the capsid in both cardiac cell types (Fig. 1A, B, right panels). This was further validated in hiPSC-CM using the top 3 cardiac-specific promoters packaged individually in non-barcoded rAAV6-GFP-expressing vectors (Fig. S1). On the other hand, cTnI was the lowest ranked for transduction across both cardiac in vitro models (Fig. 1A, B, right panels). Interestingly, NCX also demonstrated leaky expression in rAAV2-transduced HuH7 compared to cTnT (Fig. 1C, right panel), thus validating the cardiac specificity of the cTnT promoter.

### cTnT was among the top performing cardiac-specific promoter for cardiac transduction in mice with reduced liver transduction

**Fig. 2:**
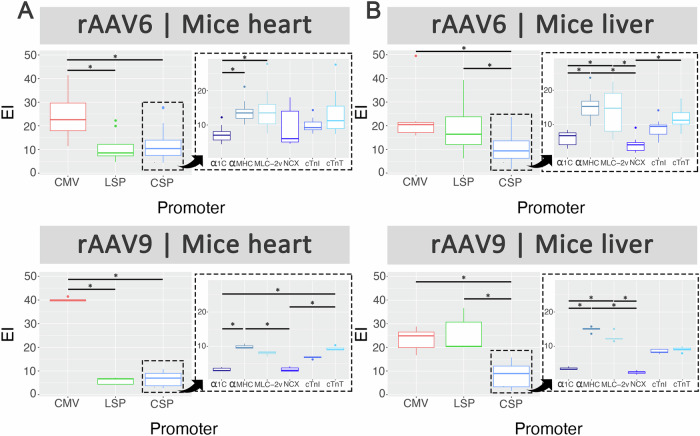
Competitive transduction assay to compare efficiency of barcoded rAAV promoters in mice. C57Bl6 mice (6–8 week male) were injected with a rAAV promoter kit packaged in AAV6 (*n* = 8) and AAV9 (*n* = 5) at 1×10^11^ vg via the tail vein. Four weeks post transduction, mice were sacrificed. **A** Heart and **B** liver tissues were collected for DNA/RNA extraction and NGS analysis. For each capsid and tissue combination, results on the left are displayed as boxplots comparing performance of ubiquitous promoter CMV, liver-specific promoter (LSP) and cardiac-specific promoters (CSP), and on the right as boxplots comparing performance of cardiac-specific promoters only. Statistical analyses were performed using the Kruskal-Wallis test followed by Dunn’s post-hoc test with Bonferroni correction (*p < 0.05).

Male mice injected systemically with the barcoded rAAV promoter kit packaged in AAV6 and AAV9 were evaluated for cardiac and hepatic transduction by qPCR (Fig. S2) and NGS analysis. As expected, the ubiquitous promoter CMV performed the best in heart tissues of mice (Fig. 2A, left panels). As for the liver, LSP outperformed the cardiac-specific promoters regardless of capsid (Fig. 2B, left panels).

There was a considerable amount of variability in promoter performance in rAAV6-transduced mice in both heart and liver tissues when compared to rAAV9-transduced animals. Despite this, αMHC showed highest level of cardiac transduction in mice for both AAV6 and AAV9 (Fig. 2A, right panels). In terms of subsequent ranking, transduction efficiency of MLC-2v and cTnT were relatively higher than the other promoters and were comparable for rAAV6- and rAAV9-injected mice.

Notably, the top three aforementioned cardiac promoters (αMHC, MLC-2v and cTnT) also showed expression in the liver regardless of capsid used (Fig. 2B, right panels). Of them, cTnT demonstrated relatively lower liver expression thus validating the comparitive cardiac specificity of the cTnT promoter. Although α1c and NCX were least active in mice liver of all cardiac-specific promoters tested, they were not highly ranked in the heart.

### cTnT was among the top performing cardiac-specific promoter for cardiac transduction in rats with reduced liver transduction

**Fig. 3:**
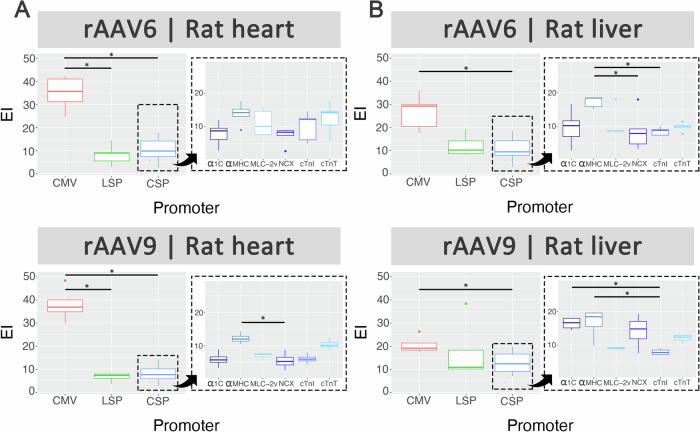
Competitive transduction assay to compare efficiency of barcoded rAAV promoters in rats. SD rats (6–8 week male) were injected with a rAAV promoter kit packaged in AAV6 (*n* = 5) and AAV9 (*n* = 4) at 5 × 10^11^ vg via the tail vein. Four weeks post transduction, rats were sacrificed. **A** Heart and **B** liver tissues were collected for DNA/RNA extraction followed by NGS analysis. For each capsid and tissue combination, results on the left are displayed as boxplots comparing performance of ubiquitous promoter CMV, liver-specific promoter (LSP) and cardiac-specific promoters (CSP), and on the right as boxplots comparing performance of cardiac-specific promoters only. Statistical analyses were performed using the Kruskal-Wallis test followed by Dunn’s post-hoc test with Bonferroni correction (*p < 0.05).

Male rats injected systemically with the barcoded rAAV promoter kit packaged in AAV6 and AAV9 were evaluated for cardiac and hepatic transduction by qPCR (Fig. S2) and NGS analysis. As expected, the ubiquitous promoter CMV performed the best in heart tissues of rats (Fig. 3A, left panels). Interestingly, there were no differences in hepatic transduction between LSP and the cardiac-specific promoters regardless of capsids (Fig. 3B, left panels).

Focusing on the performance of the cardiac-specific promoters, αMHC and cTnT showed highest level of cardiac transduction in rats for both AAV6 and AAV9, with a trend towards significance (Fig. 3A, right panels). As observed in mice, αMHC showed comparatively high hepatic expression in rats while cTnT maintained its cardiac specificity with relatively low activity in liver regardless of capsid used (Fig. 3B, right panels).

### cTnT was the top-performing cardiac-specific promoter for cardiac transduction in sheep and pigs

**Fig. 4:**
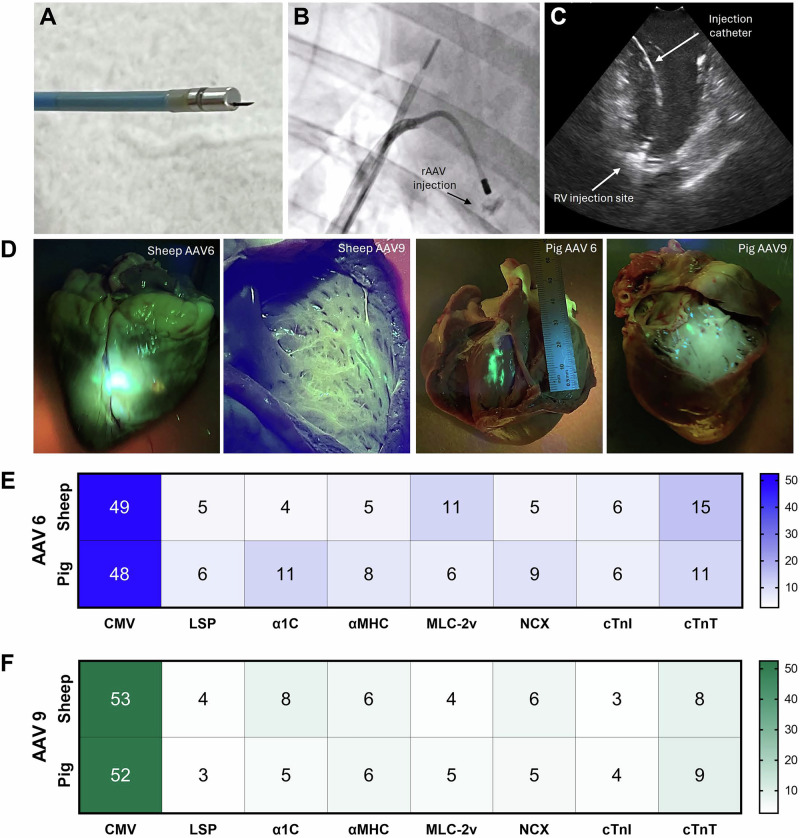
Competitive transduction assay to compare efficiency of barcoded rAAV promoters in sheep and pigs. Adult sheep and pig (*n* = 1 per capsid) were intramyocardially injected with a rAAV promoter kit packaged in AAV6 at 5 × 10^11^ vg/animal and AAV9 at 1 × 10^12^ vg/animal. **A** Endocardial gene delivery using the injection catheter with a retractable 27 gauge needle under **B** fluoroscopic and **C** intracardiac echocardiography guidance. **D** GFP fluorescence signal at RV was visualised two weeks post transduction. GFP-expressing heart tissues were collected for DNA and RNA extraction for NGS. Heat maps are expressed as expression index for **E** AAV Capsid 6 and **F** AAV Capsid 9.

Sheep and pigs injected with the rAAV promoter kit packaged in AAV6 and AAV9 were evaluated for cardiac transduction by NGS analysis. The GFP fluorescence signal at the RV septum confirmed focal transduction of cardiac tissue (Fig. 4D). The CMV promoter yielded highest level of cardiac transduction in both animal models regardless of capsids used (Fig. 4E, F). The next best promoter that produced a consistently high ranking across rAAV6- and rAAV9-injected sheep and pigs was the cTnT promoter (Fig. 4E, F). Interestingly, α1c tied with cTnT in both rAAV6-injected pig and rAAV9-injected sheep.

### cTnT showed the most promise overall as a cardiac-specific promoter

**Fig. 5:**
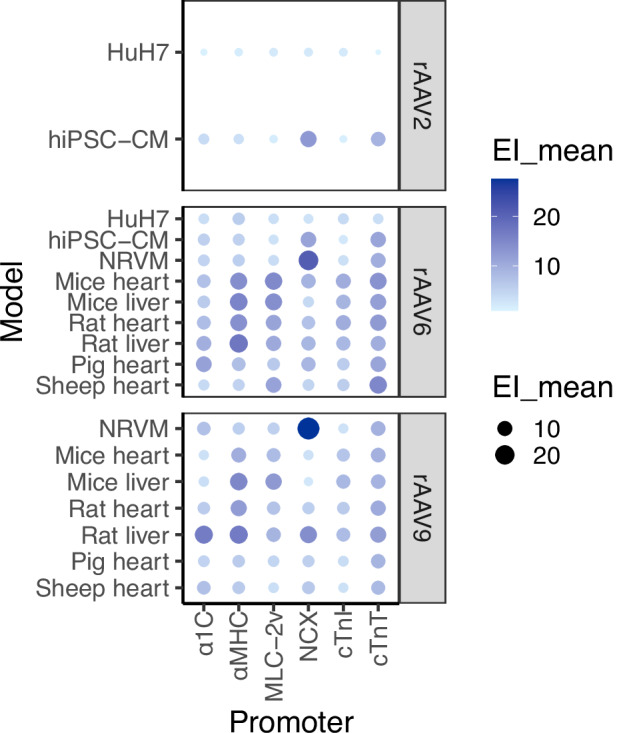
Summary of promoter efficiencies across all models tested. The size and colour of the dots indicate the mean expression intensity (EI_mean), with larger dots and darker colours corresponding to higher proportion.

NCX demonstrated the highest expression in both rAAV6- and rAAV9-transduced NRVMs. αMHC and MLC-2v show strong cardiac performance, particularly in the in vivo models, although they also exhibited considerable activity in the liver. Meanwhile, cTnT showed the most promise overall as a cardiac-specific promoter (Fig. 5).

## Discussion

This study is the first to apply a novel AAV barcode-seq method for the high-throughput evaluation of a panel of cardiac-specific promoters simultaneously. Our in vitro data demonstrated that NCX and cTnT were the best performing promoters in commonly used cardiomyocyte-enriched cell culture models tested across AAV2, AAV6 and AAV9. This finding was supported by previous research where NCX was identified as highly cardiac-specific when compared against seven individual cell lines transduced with lentiviral vectors bearing different promoters [14]. However, our data showed NCX demonstrated leaky expression in rAAV2-transduced HuH7 liver carcinoma cells relative to others, likely due to aberrant transcription factor activity in tumour cell lines. In comparison, cTnT expression was lowest ranked and almost negligible in HuH7 cells for both AAV2 and AAV6, thus validating its cardiac specificity in vitro.

Animal models used for in vivo testing provide a platform for studying the potential effectiveness of AAV-based gene therapy in a setting that mimics human cardiac disease. In the current study, we used small and large animal models for the simultaneous assessment of promoter efficiencies harnessing the barcode-seq technology. As an off-target sink, the liver can cause adverse outcomes of systemic AAV therapy. An important consideration is to assess for unintended expression in the liver when these promoters are intended to be preferentially restricted to the heart. Interestingly, we found that promoter leakiness varied between species, with αMHC exhibiting the highest hepatic leakage in both mice and rats regardless of capsid used, while α1c and NCX showed substantial liver activity in rats.

Despite these species-dependent differences, the αMHC consistently displayed robust cardiac and hepatic expression following systemic delivery of the rAAV promoter kit. Similar to our findings, it has been reported that cardiomyocyte-specific gene expression was elicited from an AAV vector harbouring an α-MHC promoter packaged in AAV2 after direct injection into myocardial wall of mice [22]. While in this case the αMHC promoter would appear to be similar in strength and specificity to the cTnT promoter that we used, the limited efficiency of the AAV2 capsid used by Aikawa et al. made direct intramyocardial injection necessary, whereas the high efficiency and natural cardiotropism of the AAV6 and AAV9 capsids supported systemic administration in our study.

While small animal models are useful for initial screening, large animals have physiology, immune responses, and anatomy that are closer to humans. Hence evaluating the safety and efficacy of cardiac promoters for AAV-based cardiac gene therapy in clinically relevant models is an important step in pre-clinical studies. Collective data from our competitive transduction studies performed in sheep and pigs exhibited highest level cardiac performance from the cTnT promoter, thus reinforcing our results from in vitro and small animal model testing. However, it should be acknowledged that hepatic transduction was not assessed in the large animal models tested and sample size was limited due to resource considerations. Furthermore, direct comparisons between the small and large animal models tested in the study should be mindfully approached since contrary to the commonly used systemic AAV delivery in pre-clinical models, we employed catheter-based intramyocardial delivery in sheep and pigs which has the potential to achieve high concentration of AAV to the heart while minimising exposure to other organs.

As one of the three subunits of the troponin (troponin C, troponin T and troponin I) found in cardiac thin filaments, cardiac troponin T (cTnT) binds to tropomyosin to form the troponin-tropomyosin complex which plays an important role in regulating contractile function. While our study lacked functional data on the efficacy of the cTnT promoter, other studies have explored this extensively. Ma et al. performed mutational analyses on the cTnT promoter region and reported that the −375 to +43 region (relative to the cTnT transcriptional start site) is sufficient to confer cardiomyocyte-specific gene expression [23]. This was further supported by bioluminescence imaging studies where it was revealed that in mice that received AAV carrying the firefly luciferase cDNA driven by this truncated cTnT promoter, luciferase expression was almost 100-fold greater in the heart than in any other tissue assayed [24]. Bezzerides et al. have also demonstrated that cTnT promoter driven AAV-mediated delivery of a CaMKII peptide inhibitor to the heart was effective in suppressing arrhythmias in a murine model of CPVT [25]. More recently, a study investigating the role of Plakophilin-2a (PKP2a) in a mouse model of ARVC showed that systemic delivery of an rAAVrh.74 vector, recognised for its preferential binding to striated muscle, in conjunction with the cardiac-specific cTnT promoter demonstrated robust transduction efficiency and an elevated expression of PKP2a specifically within the heart, with minimal expression observed in skeletal muscle and the liver [26]. This study further reinforces the importance of not only selecting an appropriate capsid but also a tissue-specific promoter to guarantee successful targeted expression of the gene of interest.

A major finding of the current study is the relatively low levels of hepatic activity of cTnT promoter independent of AAV serotype, making it a highly tissue-specific choice for cardiac gene therapy. While the application of tissue-specific promoters does not decrease viral load in the liver, other approaches to reduce hepatic transduction have been extensively explored to enable systemic gene transfer in a clinical setting. One such approach using domain swapping of the AAV2 variable region VIII with corresponding residues from other AAV serotypes such as AAV8 led to the creation of an engineered variant, AAV2i8, the first example of a liver-de-targeted vector [27]. Using AAV2i8, Hajjar and colleagues demonstrated that delivery of a constitutively active inhibitor of protein phosphatase 1, I1c, ameliorated heart failure symptoms in a porcine model of heart failure [28]. Based on these promising results this novel variant is currently being evaluated in a phase II gene therapy clinical trial for congestive heart failure [29]. Another approach is the inclusion of inhibitory miRNA binding sequences that have also been shown to reduce off-target expression, for example in the liver [30]. While our data provides convincing evidence that the cTnT promoter is most effective in our cardiac pre-clinical models tested, we cannot make conclusions about expression in the context of the human liver. This could be assessed further in a humanised-liver rodent model [31].

While this study provides valuable insights into the effects of expression cassette design on transduction efficacy, several limitations should be considered. While the potential mechanisms underlying the observed differences in promoter performance were not evaluated in this study, the complex interplay between transcription factors, chromatin modifications, enhancer-promoter interactions, and evolutionary factors that affect tissue-specific gene expression have been well documented [32–34]. The novel AAV barcode-seq method used in this study primarily offers only a relative assessment of promoter efficiency but does not provide absolute data for individual constructs, unlike demonstrated by Prasad et al. [24]. Another limitation is the potential risk of biases introduced in selecting the promoters tested which might have influenced the diversity and representation of constructs within the library. Future research with a wider scope of heterogeneous promoters including synthetic AAV promoters such as the muscle-specific SPc5-1 [35] could provide more comprehensive insights into targeted gene delivery to the heart.

## Supplementary information

Supplementary Materials and Methods

## Data Availability

The data that support the findings of this study are present in the paper and/or the Supplementary Materials. Additional raw data are available from the corresponding author upon request.

## Code availability

The code used to analyse the data and generate the study summary is provided in Supplementary Table S3.
