# Supplementary material for: High-throughput evaluation of cardiac-specific promoters for adeno-associated virus mediated cardiac gene therapy
Source: Gene Ther. 2025 Jul 19;33(2):203–10. doi: 10.1038/s41434-025-00553-6 (PMC13056562; doi:10.1038/s41434-025-00553-6)
Supplement: Supplementary file 1 — Supplementary Materials and Methods [file 41434_2025_553_MOESM1_ESM.docx]

**Supplementary Materials and Methods**

**Method S1. Construction and production of cardiac-specific barcoded rAAV promoter kit**

AAV vector plasmids containing ITR2-CMV-βglobin intron-eGFP-N_6_Barcode (BC)-WPRE-ITR2 (pAAV CMV eGFP BC WPRE) each with a unique 6-mer barcode (Table S1) were obtained from the Translational Vectorology Research Unit, Children’s Medical Research Institute, Sydney, Australia.

The CMV promoter was replaced by one of the following promoters i) ApoE hAAT Liver specific promoter (LSP), ii) L-type calcium channel α-subunit (α1C), iii) α Myosin heavy chain (αMHC), iv) Myosin ventricular light chain (MLC-2v), v) Sodium calcium Exchanger (NCX1), vi) Cardiac troponin I (cTnI), and vii) Cardiac troponin T (cTnT). In brief, the CMV promoter was excised with Kpn1 and Sac1 from pAAV CMV eGFP BC WPRE vector and either a PCR amplified- or gene block-generated promoter sequence with Kpn1 and Sac1 sites was ligated into the vector backbone to create each of the cardiac promoter constructs.

The rAAV promoter kits packaged in AAV2, 6 and 9 were purchased from the Vector Genome Engineering Facility, Children’s Medical Research Institute, Sydney, Australia. In brief, viral production was performed using the conventional adherent HEK293 cell method. For individual promoter constructs two plasmids with the same promoter but each containing a unique barcode were mixed in equimolar proportions and used for rAAV packaging. The crude preparations for each promoter were quantified and mixed at equal proportions prior to purification using cesium chloride density centrifugation. The rAAV promoter kit was concentrated, titred using qPCR.

**Method S2. Human iPSC maintenance and differentiation into cardiomyocytes (hiPSC-CMs)**

The human iPSC line SCVI 8 was obtained from Prof. Joseph Wu (Stanford Cardiovascular Institute). It was maintained on Matrigel (Corning, New York, no. 354277) coated 60 mm cell culture dishes using the mTeSR Plus kit (STEMCELL Technologies, Vancouver, Canada, no. 05825). Upon confluence, cells were passaged as colonies using gentle cell dissociation reagent (STEMCELL Technologies, no. 07174) every 6–7 days. In brief, the differentiation was commenced on D0, using the STEMdiff Cardiomyocyte Differentiation Kit according to the manufacturer’s instructions until D11, followed by 2 days of metabolic selection using lactate medium composed of glucose free DMEM (Thermo Fisher Scientific, 2A14430-01). On D13, cells were returned to STEMdiff Cardiomyocyte Maintenance Medium (CMM).

On D15, cardiomyocytes were replated into Geltrex-coated 24-well plates at 500,000 cells/well using RPMI 1640 medium (Thermo Fisher Scientific, 21870076) and B27 supplement (Thermo Fisher Scientific, 17504-001) accompanied with Y-27632. Flow cytometry was performed using cells from two wells to assess cardiomyocyte purity. Batches of cells that were greater than 90% cTnT positive were used for transduction with the rAAV promoter kit.

**Method S3. Flow cytometry to determine cardiomyocyte purity**

Cells were dissociated using TrypLE Express (Thermo Fisher Scientific, Waltham.MA, no. 12604-021), then washed with Dulbecco’s phosphate-buffered saline without calcium and magnesium (Lonza, Basel, Switzerland, no. 12001-664). Cells were then stained using the Zombie NIR Fixable Viability Kit (BioLegend, San Diego, CA, no. 423105). After washing, cells were fixed in 4% PFA (w/v) for 30 min, then washed and further stained with BV421-conjugated mouse anti-cTnT antibody (BD Biosciences, San Diego, CA, no. 565618) for 2 hours as described (19). Cells were analysed for cTnT expression on the FACS Canto II Cell Analyzer (BD Biosciences, San Jose, CA) and data recorded using FACS Diva Software (BD Biosciences, Franklin Lakes, NJ). Analysis was subsequently performed using FlowJo (FlowJo, Ashland, OR) version 10.

**Method S4. Recombinant AAV production for individual promoter vectors**

Constructs bearing the cardiac-specific promoters αMHC, NCX and cTnT, as well as controls CMV and LSP, were packaged in AAV6. Recombinant AAV production, purification, concentration and titration were carried out as described (20). In brief, 40x10cm dishes of HEK293 cells were transfected with 2 plasmids (promoter plasmid and pDGM6 rep/cap helper plasmid) with calcium phosphate. Virus was collected from the supernatant 48 hours post transfection. AAV was purified by PEG precipitation followed by caesium chloride density gradient separation. Following dialysis vector was concentrated and titred by qPCR.

**Method S5. Flow cytometry for quantification of individual promoter activity in hiPSC-CM**

Human iPSC-CM differentiations that showed greater than 90% cTnT purity by flow cytometry were transduced with each of the five individual promoter rAAVs in triplicate at an MOI of 1000. On D5 post transduction, cells were imaged using the Zeiss Axiovert 200M Live Cell Imaging Microscope. Cells were then analysed for GFP expression on FACS Canto II Cell Analyzer and data recorded using FACS Diva Software. Analysis was subsequently performed using FlowJo version 10.

**Method S6. Real-time qPCR for vector genomes quantitation in tissues from mouse and rat**

Genomic DNA was extracted from heart and liver of mice and rats using the Allprep DNA/RNA mini kit. Real-time qPCR was used to quantify vector genomes. In brief, WPRE 5’ forward (5’- CCGTTGTCAGGCAACGTG -3’) and 3’ reverse (5’- AGCTGACAGGTGGTGGCAAT -3’) primers, as well as FAM-TGCTGACGCAACCCCCACTGGT-TAMRA probe were used. Reactions were set up in a 384-well plate (Biorad, Hercules, CA, USA) and conducted in 10μL volumes using the following mix: 1×Sensifast Probe No-ROX qPCR master mix (Bioline, Memphis, TN, USA), 0.4μM primers, 0.1μM probe, 100ng of genomic DNA and molecular biology grade sterile water (Lonza, Basel, Switzerland). The plates were run in a Biorad CFX384 PCR thermocycler (Biorad, Hercules, CA, USA) using the following conditions: denaturation at 95⁰C for 5 minutes, 40 cycles of denaturing for 15 seconds at 95⁰C, annealing and extension for 35 seconds at 60⁰C. The cycle threshold values obtained from triplicate samples were converted to vector copies per 100ng genomic DNA, using a standard curve generated from serial dilutions of a rAAV.GFP linearized plasmid.

**Table S1. Details of promoter sizes, barcode numbers and barcode sequences**

| **Promoter** | **Barcode number** | **Barcode Sequence** | **Promoter Size (bp)** |
| --- | --- | --- | --- |
| **CMV** | BC 24 | CCTTCT | 584 |
|  | BC 31 | TCAACC |  |
| **LSP** | BC 40 | GGAAAA | 1065 |
|  | BC 42 | GTGTTC |  |
| **α1C** | BC 18 | ACCCAG | 1575 |
|  | BC 19 | GCAATC |  |
| **αMHC** | BC 16 | GGCACG | 393 |
|  | BC 17 | CGCGAC |  |
| **MLC-2v** | BC 2 | AAAGAC | 251 |
|  | BC 4 | ACCTTT |  |
| **NCX** | BC 11 | CCTCCG | 2059 |
|  | BC 14 | AATAGT |  |
| **cTnI** | BC 5 | ACCAAA | 246 |
|  | BC 9 | CAGGGA |  |
| **cTnT** | BC 20 | ACAAAA | 414 |
|  | BC 21 | TGATGT |  |

| **Table S2. Summary data for promoter performance expressed as expression index (EI) across various models. Data used with R script (see also Table S3) for generating Figure 5.** | | | | | | | | |
| --- | --- | --- | --- | --- | --- | --- | --- | --- |
| **rAAV2** | | | **rAAV6** | | | **rAAV9** | | |
| **Promoter** | **Model** | **EI** | **Promoter** | **Model** | **EI** | **Promoter** | **Model** | **EI** |
| α1c | HuH7 | 1.196727 | α1c | HuH7 | 2.640657 | α1c | NRVM | 5.145149 |
| α1c | HuH7 | 1.050368 | α1c | HuH7 | 5.325117 | α1c | NRVM | 6.374021 |
| α1c | HuH7 | 1.371389 | α1c | HuH7 | 3.015824 | α1c | NRVM | 11.42909 |
| αMHC | HuH7 | 1.542703 | αMHC | HuH7 | 7.35969 | α1c | NRVM | 7.09342 |
| αMHC | HuH7 | 1.784342 | αMHC | HuH7 | 6.098594 | α1c | NRVM | 7.073158 |
| αMHC | HuH7 | 2.106511 | αMHC | HuH7 | 3.788116 | αMHC | NRVM | 4.001323 |
| MLC-2v | HuH7 | 1.866554 | MLC-2v | HuH7 | 2.62961 | αMHC | NRVM | 6.047058 |
| MLC-2v | HuH7 | 1.881298 | MLC-2v | HuH7 | 4.293259 | αMHC | NRVM | 9.237585 |
| MLC-2v | HuH7 | 2.399275 | MLC-2v | HuH7 | 3.474378 | αMHC | NRVM | 3.676183 |
| NCX | HuH7 | 2.533268 | NCX | HuH7 | 2.723191 | αMHC | NRVM | 4.588747 |
| NCX | HuH7 | 1.85837 | NCX | HuH7 | 3.24856 | MLC-2v | NRVM | 7.801771 |
| NCX | HuH7 | 2.218638 | NCX | HuH7 | 2.687041 | MLC-2v | NRVM | 3.495666 |
| cTnI | HuH7 | 2.219999 | cTnI | HuH7 | 3.116366 | MLC-2v | NRVM | 9.807569 |
| cTnI | HuH7 | 1.745182 | cTnI | HuH7 | 5.562428 | MLC-2v | NRVM | 2.701382 |
| cTnI | HuH7 | 2.053941 | cTnI | HuH7 | 3.344473 | MLC-2v | NRVM | 2.917048 |
| cTnT | HuH7 | 0.730852 | cTnT | HuH7 | 2.982418 | NCX | NRVM | 31.34064 |
| cTnT | HuH7 | 0.938761 | cTnT | HuH7 | 4.5136 | NCX | NRVM | 33.68175 |
| cTnT | HuH7 | 1.135505 | cTnT | HuH7 | 3.087947 | NCX | NRVM | 19.39306 |
| α1c | hiPSC-CM | 4.16856 | α1c | hiPSC-CM | 4.670951 | NCX | NRVM | 25.46693 |
| α1c | hiPSC-CM | 4.04192 | α1c | hiPSC-CM | 4.430062 | NCX | NRVM | 27.2945 |
| α1c | hiPSC-CM | 3.287431 | α1c | hiPSC-CM | 7.208602 | cTnI | NRVM | 3.167245 |
| α1c | hiPSC-CM | 4.09352 | α1c | hiPSC-CM | 5.007464 | cTnI | NRVM | 2.735517 |
| αMHC | hiPSC-CM | 2.913996 | αMHC | hiPSC-CM | 4.814576 | cTnI | NRVM | 4.599288 |
| αMHC | hiPSC-CM | 3.192211 | αMHC | hiPSC-CM | 5.00969 | cTnI | NRVM | 2.690471 |
| αMHC | hiPSC-CM | 2.405746 | αMHC | hiPSC-CM | 6.658148 | cTnI | NRVM | 2.43238 |
| αMHC | hiPSC-CM | 4.791072 | αMHC | hiPSC-CM | 4.157586 | cTnT | NRVM | 11.63247 |
| MLC-2v | hiPSC-CM | 1.591693 | MLC-2v | hiPSC-CM | 2.420653 | cTnT | NRVM | 6.957515 |
| MLC-2v | hiPSC-CM | 1.847673 | MLC-2v | hiPSC-CM | 2.348756 | cTnT | NRVM | 7.62197 |
| MLC-2v | hiPSC-CM | 1.350311 | MLC-2v | hiPSC-CM | 4.40825 | cTnT | NRVM | 10.93024 |
| MLC-2v | hiPSC-CM | 2.500218 | MLC-2v | hiPSC-CM | 3.037661 | cTnT | NRVM | 9.360375 |
| NCX | hiPSC-CM | 14.42008 | NCX | hiPSC-CM | 10.30644 | α1c | Mice heart | 2.896993 |
| NCX | hiPSC-CM | 13.60496 | NCX | hiPSC-CM | 9.859232 | α1c | Mice heart | 2.822912 |
| NCX | hiPSC-CM | 10.37355 | NCX | hiPSC-CM | 9.674415 | α1c | Mice heart | 2.781534 |
| NCX | hiPSC-CM | 11.71905 | NCX | hiPSC-CM | 14.40876 | α1c | Mice heart | 3.686158 |
| cTnI | hiPSC-CM | 1.239948 | cTnI | hiPSC-CM | 1.883631 | α1c | Mice heart | 3.902811 |
| cTnI | hiPSC-CM | 1.294944 | cTnI | hiPSC-CM | 1.899096 | αMHC | Mice heart | 9.58649 |
| cTnI | hiPSC-CM | 0.878669 | cTnI | hiPSC-CM | 3.340604 | αMHC | Mice heart | 9.668849 |
| cTnI | hiPSC-CM | 2.48568 | cTnI | hiPSC-CM | 2.09377 | αMHC | Mice heart | 9.312708 |
| cTnT | hiPSC-CM | 8.844532 | cTnT | hiPSC-CM | 13.85384 | αMHC | Mice heart | 9.392075 |
| cTnT | hiPSC-CM | 9.14608 | cTnT | hiPSC-CM | 11.92473 | αMHC | Mice heart | 10.78836 |
| cTnT | hiPSC-CM | 7.499617 | cTnT | hiPSC-CM | 11.04259 | MLC-2v | Mice heart | 8.413065 |
| cTnT | hiPSC-CM | 10.80004 | cTnT | hiPSC-CM | 6.742492 | MLC-2v | Mice heart | 8.06288 |
|  |  |  | α1c | NRVM | 5.105239 | MLC-2v | Mice heart | 8.138756 |
|  |  |  | α1c | NRVM | 5.064456 | MLC-2v | Mice heart | 8.233685 |
|  |  |  | α1c | NRVM | 4.553842 | MLC-2v | Mice heart | 6.968532 |
|  |  |  | α1c | NRVM | 4.667344 | NCX | Mice heart | 2.988717 |
|  |  |  | α1c | NRVM | 5.430339 | NCX | Mice heart | 2.67169 |
|  |  |  | αMHC | NRVM | 5.550443 | NCX | Mice heart | 2.725167 |
|  |  |  | αMHC | NRVM | 6.166466 | NCX | Mice heart | 3.68854 |
|  |  |  | αMHC | NRVM | 4.946174 | NCX | Mice heart | 4.153137 |
|  |  |  | αMHC | NRVM | 5.383893 | cTnI | Mice heart | 7.008617 |
|  |  |  | αMHC | NRVM | 5.236531 | cTnI | Mice heart | 6.680109 |
|  |  |  | MLC-2v | NRVM | 7.315775 | cTnI | Mice heart | 6.744694 |
|  |  |  | MLC-2v | NRVM | 2.863817 | cTnI | Mice heart | 6.928872 |
|  |  |  | MLC-2v | NRVM | 3.009786 | cTnI | Mice heart | 6.160062 |
|  |  |  | MLC-2v | NRVM | 2.946976 | cTnT | Mice heart | 8.869007 |
|  |  |  | MLC-2v | NRVM | 2.803743 | cTnT | Mice heart | 8.922349 |
|  |  |  | NCX | NRVM | 19.01075 | cTnT | Mice heart | 9.007583 |
|  |  |  | NCX | NRVM | 16.20176 | cTnT | Mice heart | 10.30576 |
|  |  |  | NCX | NRVM | 16.93989 | cTnT | Mice heart | 9.472889 |
|  |  |  | NCX | NRVM | 26.46465 | α1c | Mice Liver | 3.259712 |
|  |  |  | NCX | NRVM | 25.38119 | α1c | Mice Liver | 3.169291 |
|  |  |  | cTnI | NRVM | 3.580915 | α1c | Mice Liver | 4.118722 |
|  |  |  | cTnI | NRVM | 3.519545 | α1c | Mice Liver | 3.204434 |
|  |  |  | cTnI | NRVM | 2.847036 | α1c | Mice Liver | 3.664733 |
|  |  |  | cTnI | NRVM | 3.666082 | αMHC | Mice Liver | 15.12994 |
|  |  |  | cTnI | NRVM | 3.512309 | αMHC | Mice Liver | 13.63496 |
|  |  |  | cTnT | NRVM | 9.543891 | αMHC | Mice Liver | 15.15496 |
|  |  |  | cTnT | NRVM | 7.94411 | αMHC | Mice Liver | 15.75149 |
|  |  |  | cTnT | NRVM | 8.847902 | αMHC | Mice Liver | 14.93402 |
|  |  |  | cTnT | NRVM | 11.49656 | MLC-2v | Mice Liver | 12.15224 |
|  |  |  | cTnT | NRVM | 11.07199 | MLC-2v | Mice Liver | 11.45108 |
|  |  |  | α1c | Mice heart | 5.817728 | MLC-2v | Mice Liver | 12.09663 |
|  |  |  | α1c | Mice heart | 5.062863 | MLC-2v | Mice Liver | 15.04336 |
|  |  |  | α1c | Mice heart | 7.20379 | MLC-2v | Mice Liver | 12.22098 |
|  |  |  | α1c | Mice heart | 9.971334 | NCX | Mice Liver | 2.504964 |
|  |  |  | α1c | Mice heart | 12.22808 | NCX | Mice Liver | 2.503399 |
|  |  |  | α1c | Mice heart | 6.885011 | NCX | Mice Liver | 3.046613 |
|  |  |  | α1c | Mice heart | 7.4951 | NCX | Mice Liver | 1.663386 |
|  |  |  | α1c | Mice heart | 4.416861 | NCX | Mice Liver | 2.080138 |
|  |  |  | αMHC | Mice heart | 21.14162 | cTnI | Mice Liver | 8.253367 |
|  |  |  | αMHC | Mice heart | 10.12616 | cTnI | Mice Liver | 8.126334 |
|  |  |  | αMHC | Mice heart | 11.10895 | cTnI | Mice Liver | 9.100275 |
|  |  |  | αMHC | Mice heart | 13.79123 | cTnI | Mice Liver | 9.265418 |
|  |  |  | αMHC | Mice heart | 16.95561 | cTnI | Mice Liver | 7.654974 |
|  |  |  | αMHC | Mice heart | 13.31033 | cTnT | Mice Liver | 9.038932 |
|  |  |  | αMHC | Mice heart | 13.70597 | cTnT | Mice Liver | 8.894444 |
|  |  |  | αMHC | Mice heart | 12.19781 | cTnT | Mice Liver | 10.00634 |
|  |  |  | MLC-2v | Mice heart | 27.78668 | cTnT | Mice Liver | 9.42605 |
|  |  |  | MLC-2v | Mice heart | 14.7105 | cTnT | Mice Liver | 7.923086 |
|  |  |  | MLC-2v | Mice heart | 19.84638 | α1c | Rat heart | 5.91715 |
|  |  |  | MLC-2v | Mice heart | 9.944284 | α1c | Rat heart | 5.610691 |
|  |  |  | MLC-2v | Mice heart | 14.60569 | α1c | Rat heart | 8.917076 |
|  |  |  | MLC-2v | Mice heart | 12.44359 | α1c | Rat heart | 3.43763 |
|  |  |  | MLC-2v | Mice heart | 10.46185 | αMHC | Rat heart | 12.33588 |
|  |  |  | MLC2v | Mice heart | 7.541362 | αMHC | Rat heart | 10.51692 |
|  |  |  | NCX | Mice heart | 4.946421 | αMHC | Rat heart | 11.66904 |
|  |  |  | NCX | Mice heart | 4.560615 | αMHC | Rat heart | 14.30461 |
|  |  |  | NCX | Mice heart | 5.074643 | MLC-2v | Rat heart | 7.646457 |
|  |  |  | NCX | Mice heart | 18.05772 | MLC-2v | Rat heart | 7.100076 |
|  |  |  | NCX | Mice heart | 13.91062 | MLC-2v | Rat heart | 5.91072 |
|  |  |  | NCX | Mice heart | 6.187857 | MLC-2v | Rat heart | 7.579459 |
|  |  |  | NCX | Mice heart | 5.838798 | NCX | Rat heart | 5.810561 |
|  |  |  | NCX | Mice heart | 14.40845 | NCX | Rat heart | 4.81231 |
|  |  |  | cTnI | Mice heart | 14.32439 | NCX | Rat heart | 8.75906 |
|  |  |  | cTnI | Mice heart | 7.946769 | NCX | Rat heart | 2.64098 |
|  |  |  | cTnI | Mice heart | 9.259056 | cTnI | Rat heart | 7.882928 |
|  |  |  | cTnI | Mice heart | 9.201676 | cTnI | Rat heart | 6.135508 |
|  |  |  | cTnI | Mice heart | 7.522179 | cTnI | Rat heart | 5.859751 |
|  |  |  | cTnI | Mice heart | 10.45219 | cTnI | Rat heart | 4.38935 |
|  |  |  | cTnI | Mice heart | 12.1111 | cTnT | Rat heart | 12.50881 |
|  |  |  | cTnI | Mice heart | 8.825034 | cTnT | Rat heart | 10.05844 |
|  |  |  | cTnT | Mice heart | 27.56849 | cTnT | Rat heart | 10.27521 |
|  |  |  | cTnT | Mice heart | 12.18641 | cTnT | Rat heart | 8.533239 |
|  |  |  | cTnT | Mice heart | 19.83536 | α1c | Rat Liver | 15.32689 |
|  |  |  | cTnT | Mice heart | 7.390699 | α1c | Rat Liver | 18.04719 |
|  |  |  | cTnT | Mice heart | 7.387456 | α1c | Rat Liver | 17.94683 |
|  |  |  | cTnT | Mice heart | 10.29463 | α1c | Rat Liver | 14.45445 |
|  |  |  | cTnT | Mice heart | 14.05216 | αMHC | Rat Liver | 9.776497 |
|  |  |  | cTnT | Mice heart | 9.4549 | αMHC | Rat Liver | 19.45444 |
|  |  |  | α1c | Mice Liver | 3.873872 | αMHC | Rat Liver | 19.84928 |
|  |  |  | α1c | Mice Liver | 2.936982 | αMHC | Rat Liver | 17.40133 |
|  |  |  | α1c | Mice Liver | 7.396306 | MLC-2v | Rat Liver | 8.49915 |
|  |  |  | α1c | Mice Liver | 6.984143 | MLC-2v | Rat Liver | 8.953178 |
|  |  |  | α1c | Mice Liver | 4.133525 | MLC-2v | Rat Liver | 9.184878 |
|  |  |  | α1c | Mice Liver | 8.393367 | MLC-2v | Rat Liver | 9.411492 |
|  |  |  | α1c | Mice Liver | 8.039973 | NCX | Rat Liver | 19.30658 |
|  |  |  | α1c | Mice Liver | 6.426954 | NCX | Rat Liver | 16.30132 |
|  |  |  | αMHC | Mice Liver | 18.85461 | NCX | Rat Liver | 13.28948 |
|  |  |  | αMHC | Mice Liver | 15.5356 | NCX | Rat Liver | 7.531744 |
|  |  |  | αMHC | Mice Liver | 16.11164 | cTnI | Rat Liver | 9.022546 |
|  |  |  | αMHC | Mice Liver | 23.63076 | cTnI | Rat Liver | 7.118462 |
|  |  |  | αMHC | Mice Liver | 13.14122 | cTnI | Rat Liver | 7.227983 |
|  |  |  | αMHC | Mice Liver | 9.526219 | cTnI | Rat Liver | 8.321546 |
|  |  |  | αMHC | Mice Liver | 14.98886 | cTnT | Rat Liver | 11.87214 |
|  |  |  | αMHC | Mice Liver | 11.48263 | cTnT | Rat Liver | 12.91836 |
|  |  |  | MLC-2v | Mice Liver | 22.49746 | cTnT | Rat Liver | 12.84946 |
|  |  |  | MLC-2v | Mice Liver | 20.47881 | cTnT | Rat Liver | 10.51083 |
|  |  |  | MLC-2v | Mice Liver | 18.6162 | A1c | Pig heart | 4.907621 |
|  |  |  | MLC-2v | Mice Liver | 5.566694 | αMHC | Pig heart | 5.948249 |
|  |  |  | MLC-2v | Mice Liver | 13.68202 | MLC-2v | Pig heart | 4.639235 |
|  |  |  | MLC-2v | Mice Liver | 6.668936 | NCX | Pig heart | 5.314326 |
|  |  |  | MLC-2v | Mice Liver | 15.77627 | cTnI | Pig heart | 3.828245 |
|  |  |  | MLC-2v | Mice Liver | 8.48666 | cTnT | Pig heart | 8.951411 |
|  |  |  | NCX | Mice Liver | 1.984642 | α1c | Sheep heart | 7.485355 |
|  |  |  | NCX | Mice Liver | 2.518343 | α1c | Sheep heart | 7.648619 |
|  |  |  | NCX | Mice Liver | 5.536454 | αMHC | Sheep heart | 6.170718 |
|  |  |  | NCX | Mice Liver | 9.052013 | αMHC | Sheep heart | 6.071562 |
|  |  |  | NCX | Mice Liver | 1.784878 | MLC-2v | Sheep heart | 3.587226 |
|  |  |  | NCX | Mice Liver | 3.995495 | MLC-2v | Sheep heart | 3.604123 |
|  |  |  | NCX | Mice Liver | 4.442823 | NCX | Sheep heart | 6.302762 |
|  |  |  | NCX | Mice Liver | 4.095866 | NCX | Sheep heart | 6.485581 |
|  |  |  | cTnI | Mice Liver | 7.323746 | cTnI | Sheep heart | 3.187559 |
|  |  |  | cTnI | Mice Liver | 9.301533 | cTnI | Sheep heart | 3.196295 |
|  |  |  | cTnI | Mice Liver | 9.506846 | cTnT | Sheep heart | 8.252714 |
|  |  |  | cTnI | Mice Liver | 4.709595 | cTnT | Sheep heart | 8.17682 |
|  |  |  | cTnI | Mice Liver | 9.490827 |  |  |  |
|  |  |  | cTnI | Mice Liver | 6.57178 |  |  |  |
|  |  |  | cTnI | Mice Liver | 14.11739 |  |  |  |
|  |  |  | cTnI | Mice Liver | 10.86904 |  |  |  |
|  |  |  | cTnT | Mice Liver | 11.0459 |  |  |  |
|  |  |  | cTnT | Mice Liver | 11.42518 |  |  |  |
|  |  |  | cTnT | Mice Liver | 17.60096 |  |  |  |
|  |  |  | cTnT | Mice Liver | 7.229781 |  |  |  |
|  |  |  | cTnT | Mice Liver | 9.389011 |  |  |  |
|  |  |  | cTnT | Mice Liver | 13.54191 |  |  |  |
|  |  |  | cTnT | Mice Liver | 13.37126 |  |  |  |
|  |  |  | cTnT | Mice Liver | 10.33827 |  |  |  |
|  |  |  | α1c | Rat heart | 2.846121 |  |  |  |
|  |  |  | α1c | Rat heart | 6.148298 |  |  |  |
|  |  |  | α1c | Rat heart | 9.525308 |  |  |  |
|  |  |  | α1c | Rat heart | 11.95403 |  |  |  |
|  |  |  | α1c | Rat heart | 8.745199 |  |  |  |
|  |  |  | αMHC | Rat heart | 17.69202 |  |  |  |
|  |  |  | αMHC | Rat heart | 15.19477 |  |  |  |
|  |  |  | αMHC | Rat heart | 8.9902 |  |  |  |
|  |  |  | αMHC | Rat heart | 13.03334 |  |  |  |
|  |  |  | αMHC | Rat heart | 14.17239 |  |  |  |
|  |  |  | MLC-2v | Rat heart | 15.94628 |  |  |  |
|  |  |  | MLC-2v | Rat heart | 7.359446 |  |  |  |
|  |  |  | MLC-2v | Rat heart | 7.658146 |  |  |  |
|  |  |  | MLC-2v | Rat heart | 14.57996 |  |  |  |
|  |  |  | MLC-2v | Rat heart | 10.11293 |  |  |  |
|  |  |  | NCX | Rat heart | 2.687374 |  |  |  |
|  |  |  | NCX | Rat heart | 8.791702 |  |  |  |
|  |  |  | NCX | Rat heart | 8.341988 |  |  |  |
|  |  |  | NCX | Rat heart | 9.015732 |  |  |  |
|  |  |  | NCX | Rat heart | 7.420117 |  |  |  |
|  |  |  | cTnI | Rat heart | 6.074845 |  |  |  |
|  |  |  | cTnI | Rat heart | 5.0252 |  |  |  |
|  |  |  | cTnI | Rat heart | 12.34187 |  |  |  |
|  |  |  | cTnI | Rat heart | 14.57205 |  |  |  |
|  |  |  | cTnI | Rat heart | 12.18813 |  |  |  |
|  |  |  | cTnT | Rat heart | 10.4776 |  |  |  |
|  |  |  | cTnT | Rat heart | 5.589032 |  |  |  |
|  |  |  | cTnT | Rat heart | 14.15939 |  |  |  |
|  |  |  | cTnT | Rat heart | 17.60881 |  |  |  |
|  |  |  | cTnT | Rat heart | 14.55864 |  |  |  |
|  |  |  | α1c | Rat Liver | 2.840921 |  |  |  |
|  |  |  | α1c | Rat Liver | 16.66377 |  |  |  |
|  |  |  | α1c | Rat Liver | 11.64837 |  |  |  |
|  |  |  | α1c | Rat Liver | 10.18359 |  |  |  |
|  |  |  | α1c | Rat Liver | 6.970339 |  |  |  |
|  |  |  | αMHC | Rat Liver | 18.60175 |  |  |  |
|  |  |  | αMHC | Rat Liver | 15.87827 |  |  |  |
|  |  |  | αMHC | Rat Liver | 15.05639 |  |  |  |
|  |  |  | αMHC | Rat Liver | 18.40486 |  |  |  |
|  |  |  | αMHC | Rat Liver | 18.41047 |  |  |  |
|  |  |  | MLC-2v | Rat Liver | 18.06571 |  |  |  |
|  |  |  | MLC-2v | Rat Liver | 8.682496 |  |  |  |
|  |  |  | MLC-2v | Rat Liver | 8.702307 |  |  |  |
|  |  |  | MLC-2v | Rat Liver | 8.378466 |  |  |  |
|  |  |  | MLC-2v | Rat Liver | 8.030311 |  |  |  |
|  |  |  | NCX | Rat Liver | 3.307444 |  |  |  |
|  |  |  | NCX | Rat Liver | 17.98667 |  |  |  |
|  |  |  | NCX | Rat Liver | 9.231966 |  |  |  |
|  |  |  | NCX | Rat Liver | 7.882257 |  |  |  |
|  |  |  | NCX | Rat Liver | 4.750985 |  |  |  |
|  |  |  | cTn1 | Rat Liver | 7.382565 |  |  |  |
|  |  |  | cTnI | Rat Liver | 7.329831 |  |  |  |
|  |  |  | cTnI | Rat Liver | 8.774705 |  |  |  |
|  |  |  | cTnI | Rat Liver | 8.993146 |  |  |  |
|  |  |  | cTnI | Rat Liver | 9.979416 |  |  |  |
|  |  |  | cTnT | Rat Liver | 10.14698 |  |  |  |
|  |  |  | cTnT | Rat Liver | 7.774651 |  |  |  |
|  |  |  | cTnT | Rat Liver | 11.37882 |  |  |  |
|  |  |  | cTnT | Rat Liver | 9.575942 |  |  |  |
|  |  |  | cTnT | Rat Liver | 10.0835 |  |  |  |
|  |  |  | αA1c | Pig heart | 11.34322 |  |  |  |
|  |  |  | αMHC | Pig heart | 7.697487 |  |  |  |
|  |  |  | MLC-2v | Pig heart | 6.249582 |  |  |  |
|  |  |  | NCX | Pig heart | 8.693141 |  |  |  |
|  |  |  | cTnI | Pig heart | 5.802554 |  |  |  |
|  |  |  | cTnT | Pig heart | 10.92658 |  |  |  |
|  |  |  | α1c | Sheep heart | 4.087712 |  |  |  |
|  |  |  | α1c | Sheep heart | 4.103224 |  |  |  |
|  |  |  | αMHC | Sheep heart | 5.06539 |  |  |  |
|  |  |  | αMHC | Sheep heart | 5.140213 |  |  |  |
|  |  |  | MLC-2v | Sheep heart | 11.2779 |  |  |  |
|  |  |  | MLC-2v | Sheep heart | 11.17177 |  |  |  |
|  |  |  | NCX | Sheep heart | 4.907574 |  |  |  |
|  |  |  | NCX | Sheep heart | 4.796437 |  |  |  |
|  |  |  | cTnI | Sheep heart | 5.495286 |  |  |  |
|  |  |  | cTnI | Sheep heart | 5.573771 |  |  |  |
|  |  |  | cTnT | Sheep heart | 15.08816 |  |  |  |
|  |  |  | cTnT | Sheep heart | 14.92282 |  |  |  |

**Table S3. Script for ggplot used to generate Figure 5. See also Table S2 for data.**

| # install required packages that aren't already present  list.of.packages <- c("tidyverse", "readxl", "here")  new.packages <- list.of.packages[!(list.of.packages %in% installed.packages()[,"Package"])]  if(length(new.packages)) install.packages(new.packages) |
| --- |
| # load libraries  library(tidyverse)  library(readxl) |
| # Read data from Excel sheet  rAAV2 <- readxl::read_excel("Fig5_Data Table S2.xlsx", range="A2:C44") %>%  mutate(lib = "rAAV2")  rAAV6 <- readxl::read_excel("Fig5_Data Table S2.xlsx", range="D2:F248") %>%  mutate(lib = "rAAV6")  rAAV9 <- readxl::read_excel("Fig5_Data Table S2.xlsx", range="G2:I158") %>%  mutate(lib = "rAAV9") |
| # Order for axes  order_models <- rev(c("HuH7", "hiPSC-CM", "NRVM", "Mice heart","Mice liver", "Rat heart","Rat liver", "Pig heart", "Sheep heart"))  order_promoter <- c("α1C", "αMHC", "MLC-2v", "NCX", "cTnI", "cTnT")  # Combine data  data <- bind_rows(rAAV2, rAAV6, rAAV9) %>%  mutate(Model = factor(Model, levels = order_models)) %>%  mutate(Promoter = factor(Promoter, levels = order_promoter)) |
| # Make plot  ggplot(data, aes(x = Promoter, y = Model)) +  geom_point(aes(size = EI, color = EI)) +  facet_grid(rows = vars(lib), scales = "free") +  scale_colour_gradient(low = "#dbf3ff", high = "#003399") + # Color gradient  theme_bw(base_size = 20) +  theme(axis.text.x = element_text(angle = 90, vjust = 0.5, hjust = 1),  axis.line = element_line(color = 'black'),  plot.background = element_blank(),  panel.grid.major = element_blank(),  panel.grid.minor = element_blank()) +  labs(x = "Promoter", y = "Model") |
| # Save plot  ggsave("Figure_1.pdf", height = 10, width = 10) |


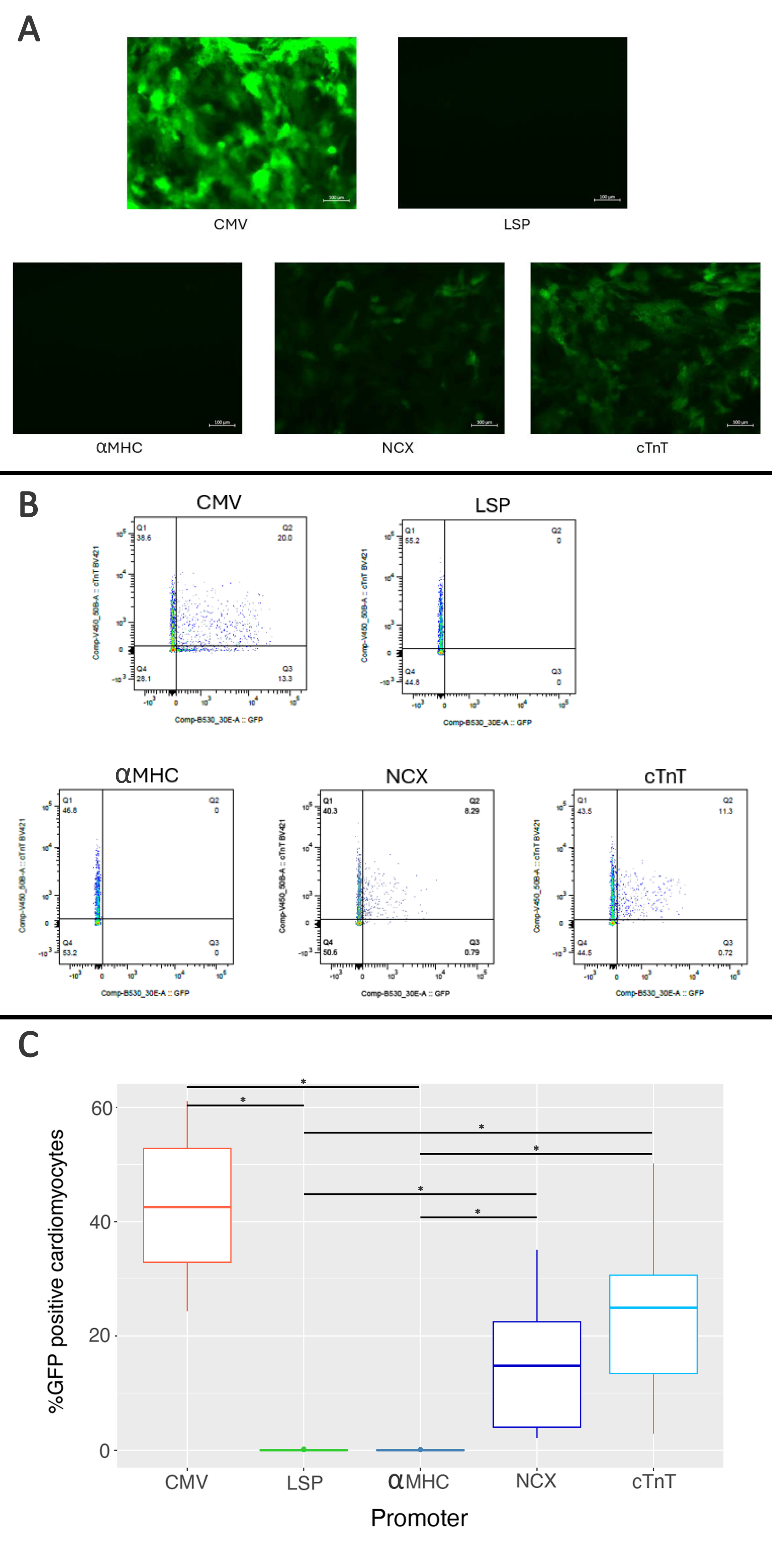


**Figure S1. Validation of individual promoter efficiencies in hiPSC-CMs.** hiPSC-CMs were transduced with the top 3 cardiac-specific promoters (αMHC, NCX and cTnT) and controls (CMV and LSP) packaged individually in non-barcoded rAAV6-GFP expressing vectors at MOI 1000, in triplicate (n=8). (A) Representative fluorescence images showing GFP auto-fluorescence in live cells at D5 post transduction. Scale bars, 100 μM. (B) Representative flow cytometry gating strategy and (C) quantification of proportions of GFP-positive cardiomyocytes (cTnT^+^ cells) in hiPSC-CMs at D5 post transduction. Statistical analyses were performed using the Kruskal-Wallis test followed by Dunn’s post-hoc test with Bonferroni correction (*p<0.05).


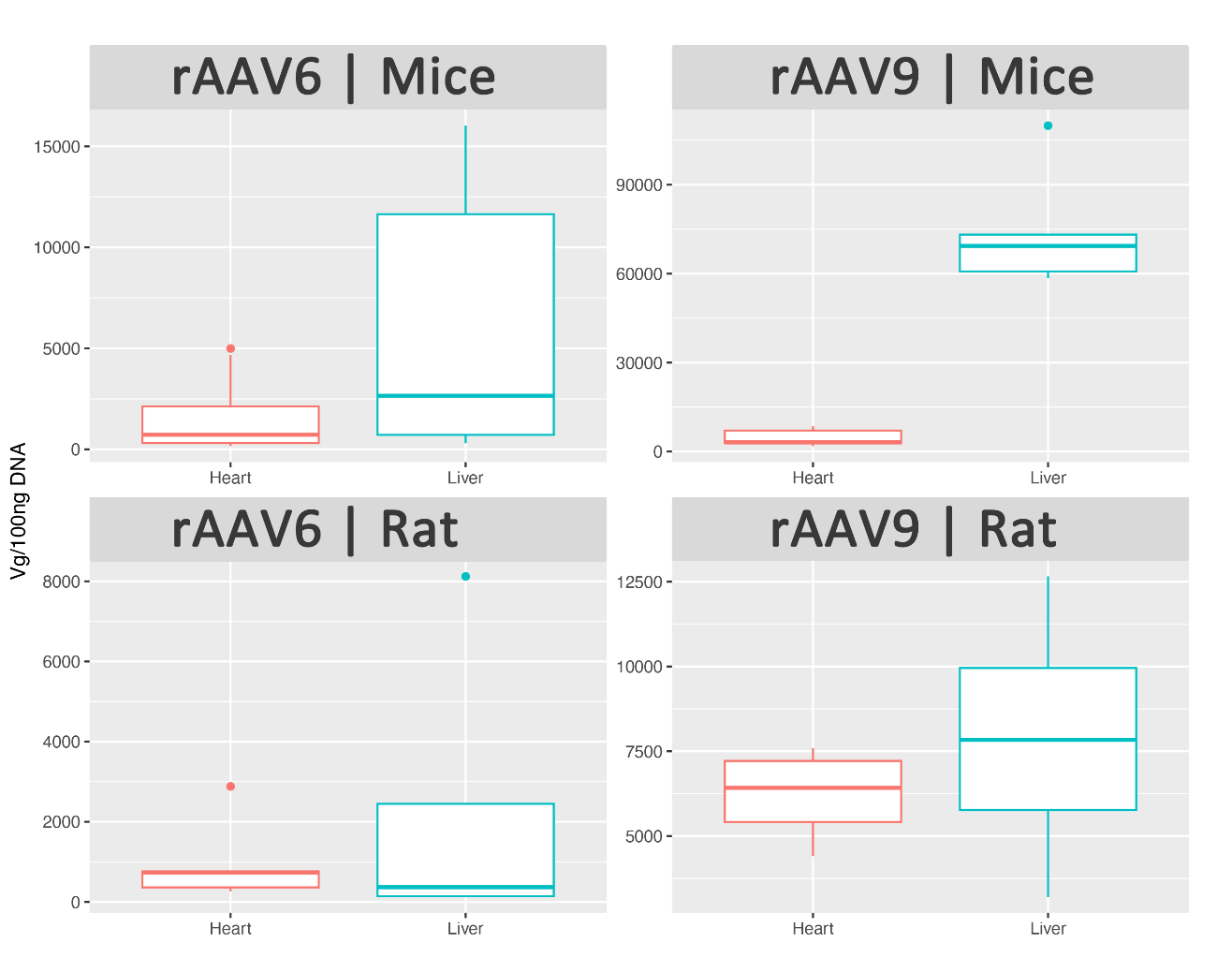


**Figure S2. Biodistribution of rAAV vector in mouse and rat according to AAV capsid.** C57Bl6 mice and SD rats (all 6-8 week male) were injected with a rAAV promoter kit packaged in AAV6 (mice n=8, rats n=5) and AAV9 (mice n=5, rats n=4) via the tail vein. Four weeks post transduction, animals were sacrificed. Heart and liver tissues were collected for DNA/RNA extraction followed by real-time qPCR. Results are displayed as boxplots comparing vector genomes per 100 ng input DNA in rAAV6- and rAAV9-transduced tissues.
